# Processing High-Solid and High-Ammonia Rich Manures in a Two-Stage (Liquid-Solid) Low-Temperature Anaerobic Digestion Process: *Start-Up and Operating Strategies*

**DOI:** 10.3390/bioengineering7030080

**Published:** 2020-07-25

**Authors:** Prativa Mahato, Bernard Goyette, Md. Saifur Rahaman, Rajinikanth Rajagopal

**Affiliations:** 1 Sherbrooke Research and Development Center, Agriculture and Agri-Food Canada, 2000 College Street, Sherbrooke, QC J1M 0C8, Canada; p_mahato@encs.concordia.ca (P.M.); bernard.goyette@canada.ca (B.G.); 2 Department of Building, Civil and Environmental Engineering, Concordia University, Montreal, QC H3G 1M8, Canada; saifur.rahaman@concordia.ca

**Keywords:** ammonia inhibition, chicken manure, dairy cow manure, high-solids anaerobic digestion, inoculum adaptation, volatile fatty acids

## Abstract

Globally, livestock and poultry production leads to total emissions of 7.1 Gigatonnes of CO_2_-equiv per year, representing 14.5% of all anthropogenic greenhouse gas emissions. Anaerobic digestion (AD) is one of the sustainable approaches to generate methane (CH_4_) from manure, but the risk of ammonia inhibition in high-solids AD can limit the process. Our objective was to develop a two-stage (liquid–solid) AD biotechnology, treating chicken (CM) + dairy cow (DM) manure mixtures at 20 °C using adapted liquid inoculum that could make livestock farming more sustainable. The effect of organic loading rates (OLR), cycle length, and the mode of operation (particularly liquid inoculum recirculation-percolation mode) was evaluated in a two-stage closed-loop system. After the inoculum adaptation phase, aforementioned two-stage batch-mode AD operation was conducted for the co-digestion of CM + DM (Total Solids (TS): 48–51% and Total Kjeldahl Nitrogen (TKN): 13.5 g/L) at an OLR of 3.7–4.7 gVS/L.d. Two cycles of different cycle lengths (112-d and 78-d for cycles 1 and 2, respectively) were operated with a CM:DM mix ratio of 1:1 (*w*/*w*) based on a fresh weight basis. Specific methane yield (SMY) of 0.35 ± 0.11 L CH_4_g/VS_fed_ was obtained with a CH_4_ concentration of above 60% for both the cycles and Soluble Chemical Oxygen Demand (CODs) and volatile solid (VS) reductions up to 85% and 60%, respectively. For a comparison purpose, a similar batch-mode operation was conducted for mono-digestion of CM (TS: 65–73% and TKN: 21–23 g/L), which resulted in a SMY of 0.52 ± 0.13 L CH_4_g/VS_fed_. In terms of efficiency towards methane-rich biogas production and ammonia inhibitions, CM + DM co-digestion showed comparatively better quality methane and generated lower free ammonia than CM mono-digestion. Further study is underway to optimize the operating parameters for the co-digestion process and to overcome inhibitions and high energy demand, especially for cold countries.

## 1. Introduction

In the last few decades, rapid growth in the population has been observed, which is further predicted to increase to 9.6 billion by 2050 [1]. In addition, the accelerated pace of urbanization and growing income is also noticed. Together, these factors pose severe challenges to the food and agriculture sectors. Along with the change in food habits, the elevation in manufactured agriculture products, mostly based on animal sources, the consumption of chicken meat, and egg production, has also increased by 50% and 36.5%, respectively, from 2000 to 2014 [2]. The demand for food is estimated to increase to 73% and 58% for meat and milk, respectively, by 2050. Consequently, this leads to mass production of livestock and, ultimately, a huge generation of manure. Manure causes emissions of greenhouse gases (GHG) [3] if not managed properly. Globally, the poultry-related emissions alone account for about 600 million tons of carbon dioxide (CO_2_) equivalent per year [4], contributing to climate change and global warming. In order to manage the manure, one of the widely exercised solutions is the land application as it provides nutrition to the land. However, excessive land application of manure results in nutrition overloading in soil and water bodies, ending up in eutrophication [5]. Open land application of manure also contributes to methane (CH_4_) emissions, which carries 23 times more global warming potential than CO_2_ alone [6]. Another positive solution towards manure management can be composting as it reduces waste mass and produces valuable end products [7]; however, the huge loss of nitrogen (N) in the form of soluble nitrates is observed in composting, which eventually reduces the fertilizer value. Besides this, composting also causes odor nuisance and environmental side effects like air and water pollution, gases like NH_3_, CH_4_, and N_2_O impacts air quality and, leaching and runoff due to precipitation causes high adverse effect on water pollution [8,9].

Anaerobic digestion (AD) is a sustainable approach to reduce the ill effects caused by improper processing of manure. In recent years, AD has received great attention due to its obvious advantage, i.e., reducing pollution, converting organic waste into high-quality biogas, which is useful in the form of heat and/or electricity [10]. Moreover, the generation of electricity through AD is a renewable process, thus reduces the cost of fossil fuels and their climatic side effects. Poultry litter is one of the highest biomethane potential organic substrates compared to dairy cow manure (DM). However, one of the major limitations of AD of chicken manure (CM) is the inhibition caused by the production of ammonia [11] due to which its potentiality cannot fully be exploited. CM is also high in solids (63 ± 10% Total Solids (TS)), which makes the process unsuitable in semi-liquid (10–15% TS) or wet (<10%) digesters as the dilution requirement would be 6–7 times than the normal practice to operate in these type of digesters. Similarly, high N in CM (Total Kjeldahl Nitrogen (TKN): 25–35 g/L) also demands huge dilution to avoid inhibitions during the AD process. Unfortunately, dilution by water requires comparatively high energy input, which makes the situation expensive and impractical to process the feedstocks rich in high-solids and high-ammonia. In this scenario, the co-digestion of CM with other crops or C-rich feedstocks could be a feasible method.

C/N ratio of CM ranges from 6.3 to 10 [12,13], and to operate the digester to its utmost condition, high carbon content is essential. On the other hand, the C/N ratio of DM is reported to be between 24 and 40 [12,14]. Therefore, co-digestion stabilizes the C:N ratio because of the composition of high lignocellulosic compounds in DM. Co-digestion also minimizes the risk of ammonia inhibitions and, in some cases, improves the methane content in the biogas. Co-digestion of manure also benefits in many ways, like the reduction of manpower in the segregation of waste to be processed. It avoids the separate storage, treatment, and handling of mixed waste [15]. Agriculture and Agri-Food Canada (AAFC) has successfully developed the AD biotechnology over the years to process poultry, swine, and cow manures operating at low temperatures. However, the potential of digesters to process the co-digestion of DM and CM at a TS > 50% using adapted liquid inoculum has not been studied. The positive results obtained from the study of the mono-digestion of CM has encouraged us to explore the possibilities of co-digesting CM + DM mix in an economical way.

This paper emphasized on the start-up and operating strategies for the development of low-temperature two-stage (liquid–solid) anaerobic co-digestion of CM + DM mixture using adapted inoculum. The primary objective of this study was to demonstrate the operational feasibility of two-stage process (i.e., liquid inoculum reservoir coupled with high-solid anaerobic digestion (HSAD) system), treating CM + DM at 20 ± 1 °C, and to encourage small-scale farmers to adopt this technology at low cost. An effort was made to develop the HSAD start-up protocol, using (i) acclimatized liquid inoculum since obtaining a huge quantity of solid inoculum to treat high-solids waste mix is practically not feasible at many farm locations; (ii) no mixing conditions, as mechanical mixers create complexity in full-scale operations. Besides, the scope of this study was also to assess the comparative performance of digesters co-digesting CM + DM and mono-digesting CM, especially in terms of methane concentration and free ammonia inhibition.

## 2. Materials and Methods

### 2.1. Feedstock and Inoculum

Fresh DM was obtained from the AAFC dairy farm located at our Sherbrooke Research and Development Center, whereas the fresh CM was sourced from a small-sized poultry farm located in Farnham (Quebec province). DM consisted of straw as bedding, whereas the bedding of CM composed of wood shavings. These bedding materials were used for the dairy cow/chicken productions by the farm itself. Hence, the manure used in this study was always contained in the bedding components. The manure was collected and stored in a cold room at 4 °C to prevent biological activity prior to feeding. For the feedstock characterization, manure was diluted and ground primarily to reduce the feed concentration for the analysis purpose and the homogenization of the solid samples, except for the TS and volatile solid (VS) analysis. The liquid inoculum used in the start-up phase was obtained from our ongoing laboratory-scale liquid sequencing batch AD, adapted to high-ammonia content chicken manure leachate. The summary of the feedstock and inoculum materials used is shown in Table 1.

### 2.2. Experimental Setup of Two-Stage (Liquid–Solid) Anaerobic Digesters

**Figure 1:**
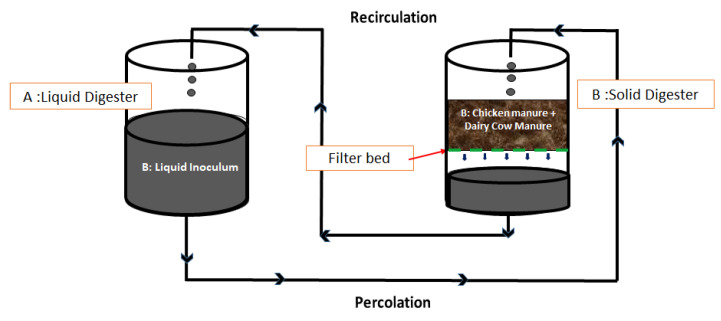
Schematic diagram of a single set of two-stage (liquid–solid) digesters.

The experimental arrangement consisted of two-stage (liquid–solid) anaerobic digesters (i.e., liquid inoculum reservoir coupled with HSAD system) for processing CM + DM mixture at 20 ± 1 °C. Two sets of digesters in duplicates with a total volumetric capacity of 40 L were operated in parallel. A set consisted of 2 digesters—one for liquid inoculum reservoir named “digester A”, and the other for HSAD named “digester B”. Digesters A and B were kept adjacent to each other, as shown in Figure 1. Therefore, the two sets of digesters were named as digester 1 (1A + 1B) and digester 2 (2A + 2B).

The concept behind this coupled liquid–solid digesters arrangement was to enhance the digestion feasibility of the HSAD content, which was fed without any dry inoculum. Provisions made in such a way that a known volume of adapted liquid inoculum from ‘digester A’ was recirculated-percolated through the solid content in the HSAD (digester B), principally (i) to enhance mixing and, thus, waste-microbe interactions in ‘digester B’ and (ii) also to leach out a significant amount of Volatile Fatty Acids (VFA) and nitrogen from digesters ‘B’ to ‘A’. By doing this, organic and VFA overloading in HSAD were minimized, but, at the same time, methane yield was increased since ‘digester A’ also contributed to producing biogas as it contained acclimatized microbes. This conception also aimed to increase the buffering capacity of the digesters by maintaining optimum pH and alkalinity in ‘digester B’. Similarly, the liquid inoculum ‘digester A’, which was less in organic matter (Table 2), got fed and charged from ‘digester B’, aiding in additional methane production.

The digesters (A and B) were fit with the biogas pipeline to the tip tank for the release and quantification of the biogas produced. Digester A was connected with 3 additional pipelines; first one was connecting A and B; second, was linked to the pump for mixing. Mixing was done (just in digester A) every day for 5 min, mainly to homogenize the liquid content since it received leachate from digester B and also to release the space for air bubbles trapped in the anaerobic digesters. Similarly, the third one was connected to B, for recirculating the liquid inoculum from B to A. The first and the third pipe connections were responsible for percolation and recirculation of liquid inoculum. Five liters of inoculum from digester ‘A’ were recirculated to digester ‘B’ and then percolated back from digesters ‘B’ to ‘A’, thrice a week.

Embracing this set-up, altogether two batch feeding operations were conducted one after the other immediately; hence, they are named as “cycle 1” and “cycle 2”, which represents retention time or treatment duration corresponding to each feeding. Cycle 1 was conducted for 112 days, while cycle 2 was conducted for 78 days only. The operation time or cycle length was mainly dependent upon the desirable methane concentration, methane yield, and VFAs accumulations. CM and DM were mixed in 1:1 (*w*/*w*) ratio for two reasons: (i) to operate the digester with TS of around 50% (instead of about 70% in CM); (ii) to maintain Total Chemical Oxygen Demand (COD_t_)/TKN ratio in the range of 30. However, further study is underway in order to optimize several operating parameters, including COD_t_/TKN ratio, as our prime aim is to operate at high ammonia levels. As presented in Table 1, a total of 7 kg of mixed manure was fed to the (HSAD) digester (cycle 1 operation). For cycle 2, about 4.7 kg of digested material resulted from cycle 1 was retained in the HSAD as a source of dry inoculum. This was done in order to reduce the start-up period by supplying adopted active microbes for the subsequent (batch) feeding. Our motive was to operate at short cycle length and to maintain a similar volumetric loading rate. Henceforth, about 4.7 kg of mixed (CM + DM) manure (refer Table 1) was mixed to the retained dry inoculum (i.e., 4.7 kg) and fed to the HSAD in order to have the substrate:dry inoculum ratio (*w*/*w*) close to 1:1. Once the stabilization occurred, substrate:dry inoculum ratio would be increased to accommodate more feedstocks for commercial benefits.

It is to be noted that the liquid inoculum used in cycle 1 was adapted to CM leachate with 5500 mg TKN/L. Since the adapted inoculum was not exposed to DM, longer retention time was required for cycle 1 operation to develop an acclimatized inoculum for cycle 2 operation. A volume of 25 L liquid inoculum was fed in the individual liquid digesters in both cycle 1 and cycle 2. The substrate to liquid inoculum digester volumetric ratio was maintained between 1:3.6 and 1:2.6 for cycles 1 and 2, respectively. The solid content of the mix was initiated with approximately 48% TS in cycle 1 and 51% TS in cycle 2.

A similar experimental set-up was used for CM mono-digestion. Two operational cycles (70-d for cycle 1 and 85-d for cycle 2) were conducted in order to have a performance comparison. Mono-digestion of CM was processed with the 65–73% TS, 4.3–4.6 gVS/L.d, and the co-digestion (CM + DM) was treated with 48–51% TS, 3.7–4.7 gVS/L.d. Two cycles of different cycle lengths, depending upon the consumption of VFAs, methane quality, and digester’s stability factors, were carried out. The operating conditions of all the four processes (CM(C1), CM + DM (C1), CM(C2), and CM + DM(C2)) are shown in Table 2.

### 2.3. Analytical Methods

The bio-digesters were operated in a batch mode; therefore, the operational physio-chemical parameters were examined only for the liquid digesters on a weekly basis in order to assess the performance of the two-stage digesters. About 100 mL liquid samples were withdrawn from the liquid inoculum reservoir for the physiochemical analysis, whereas samples from the HSAD system was only taken twice *viz.* at the beginning and the end of operation since the HASD was not having weekly sampling provisions. Overall, 290 samples for biogas and 80 samples for physiochemical tests were taken during 190 days of the entire process of CM + DM. For CM alone, 240 gas samples and 50 samples were taken during 155 days of operation.

#### 2.3.1. Biogas Analysis

The biogas production and its composition were checked for both A and B digesters on alternative days. The biogas samples were analyzed thrice a week (weekends not included from all the 4 bio-digesters (1A, 1B, 2A, and 2B)), and the volume of biogas was monitored every day using the wet tip gas meters. Methane concentration in the biogas was analyzed using a gas chromatograph (Micro GC 490, Agilent Technologies, Santa Clara, CA, USA) equipped with a thermal conductivity detector (TCD) and Helium gas as the carrier gas at a flow rate of 20 mL/min. The injector and oven temperatures were 110 °C and 180 °C, respectively.

#### 2.3.2. Physiochemical Analysis

All the other samples were analyzed for the tests like pH, alkalinity, total solids (TS), volatile solids (VS), total COD (TCOD), soluble COD (CODs), TKN, ammonia nitrogen, and volatile fatty acids (VFAs). Along with this, TS and VS on a dry weight basis were determined following the guidelines given by the standard methods [16]. pH was determined by using pH Mettler Toledo AG 8603, SevenMulti (Schwerzenbach, Switzerland). Alkalinity was measured using Hach Lagne Sarl, Titralab AT1000 Series (Hach, Switzerland). COD was measured by using a closed reflux colorimetric method [16]. TKN and NH_3_-N were analyzed using a 2460 Kjeltec Auto-Sampler System (FOSS, Sweden) following the macro-Kjeldahl method [16]. VFA was determined using a Perkin Elmer gas chromatograph, model Clarus 580 (Perkin Elmer, Shelton, CT, USA), mounted with a DB-FFAP high-resolution column, but before the evaluation of VFAs, samples were conditioned according to the procedures mentioned by Masse et al. (2003) [17]. Samples collected from digesters were first centrifuged at 41× *g* for 15 min and filtered through a 0.22 µm membrane before injected. The injection volume was 0.1 µL.

## 3. Results and Discussion

### 3.1. Characteristics of the Feedstock and the Inoculum

The characteristics of the inoculum and feedstocks are shown in Table 3. DM had low carbon in terms of CODt (~65% less) and nitrogen content in terms of TKN (~70% less) than CM, which complemented the DM to achieve a desirable nutrient content in the system for AD of CM + DM. The COD_t_/TKN ratio of the CM + DM mixture in this study was around 30, which is considered as optimum value, as reported in [18]. However, for inoculum, this ratio was low in the range of 2–3, as it was acclimatized using high ammonia content wastes. The pH of CM, DM, or CM + DM mixture was always above 7.5, although high VFA concentrations of 11.6 g/L were detected for CM, mostly because of the high amount of alkalinity in the respective manures (Table 3). The biodegradability of CM, DM, and CM + DM mixture was generally higher (i.e., VS/TS = 86–89%).

### 3.2. Influence of Operational Parameters in the Two-Stage AD Process Treating CM + DM Mixture

Two-sets of two-stage (liquid inoculum reservoir coupled with HSAD) AD digesters, treating CM + DM mixture, were operated for a total period of 190 days, in which cycle 1 was operated for 112 days (i.e., day 0–112), and then cycle 2 was done for 78 days (i.e., from day 113–190). Digester’s performance was monitored by a wide range of several physicochemical parameters listed under Section 2.3.2, in order to develop a start-up solid-state AD protocol, using adapted liquid inoculum as a microbial source. Operational parameters, such as Organic Loading Rate (OLR), cycle length/treatment period, operating temperatures, recirculation-percolation rate and frequency, and the mode of operation, were controlled as they have a direct influence on the performance of the two-stage AD process. In addition to this, the effect of ammonia concentrations on the digester’s performance was also given priority.

#### 3.2.1. Performance of the Two-Stage AD at Different Cycles and OLRs: Biogas and Methane Production and Digester Buffering Indicators

The task of liquid inoculum reservoirs was not just limited to the dilution of solid digesters organic content or to supply active microbes but also played a vital role in providing signals of the ongoing metabolic activity in the HSAD. The indications from liquid digesters assisted in taking the required actions prior to the possible inhibitions that could occur in the system. Liquid digesters also participated in the generation of biogas in addition to the HSAD with a supply of new feed from each time the leachate was recirculated.

**Figure 2:**
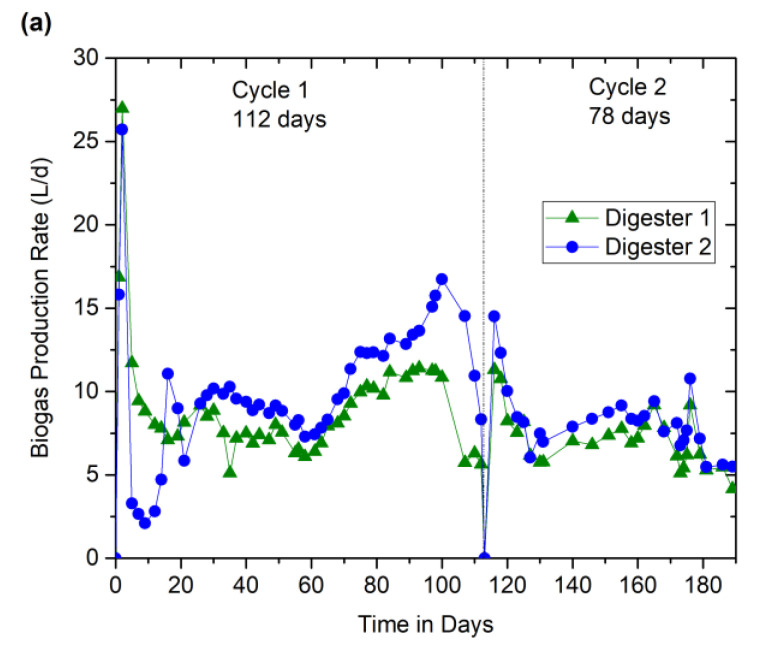

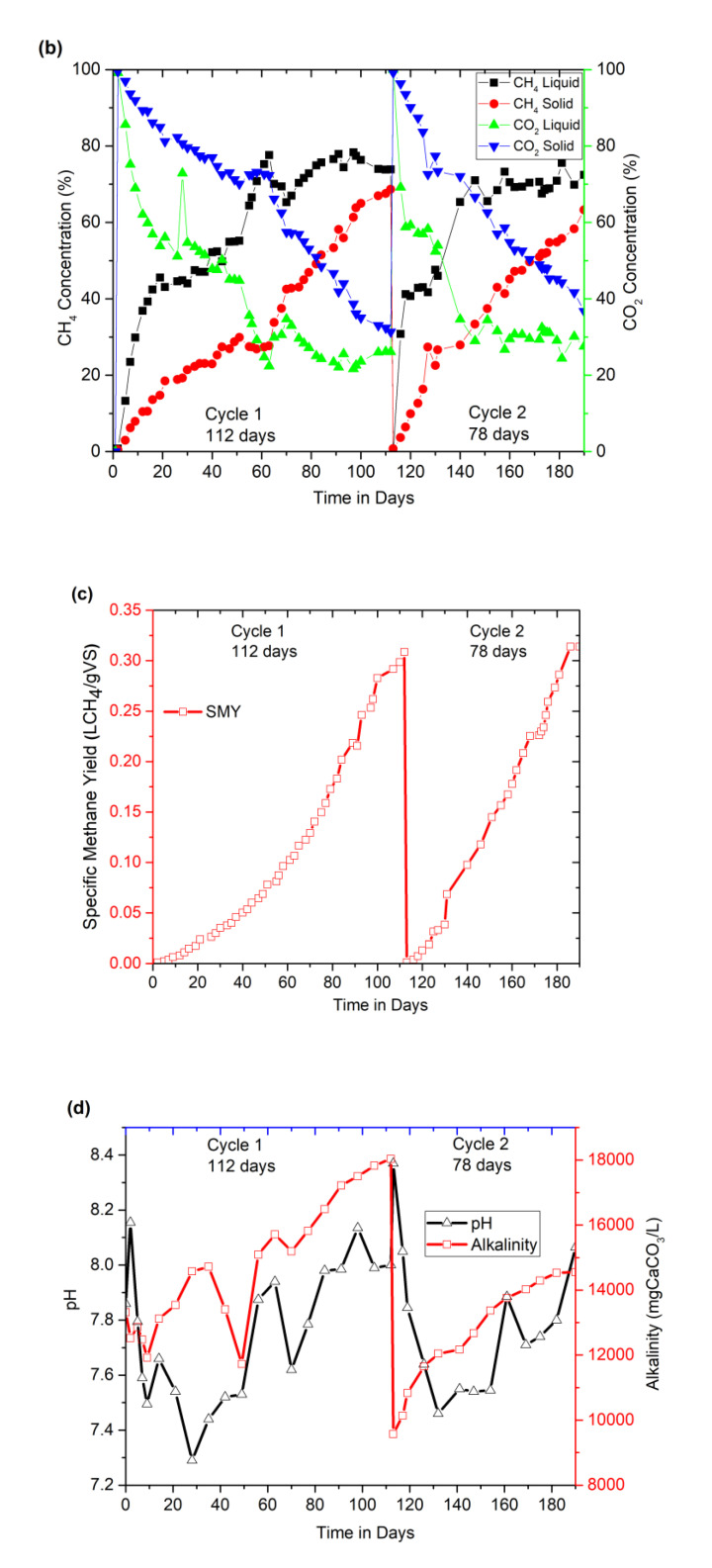
Performance of the liquid and solid digesters at different organic loading rates (OLRs) during 190 days of operation. (**a**) Biogas production rate; (**b**) Biogas composition; (**c**) Specific methane yield (SMY); (**d**) pH and alkalinity profiles.

Figure 2a–c depict the biogas and methane production profiles and their yield along with the digester buffering indicators (pH and alkalinity). For cycle 1 (days 0–112) operation, the OLR was maintained at 3.7 g.VS/L.d, and the corresponding volumetric combined (liquid + HSAD) biogas production recorded was 9.4 ± 3.7 L/d (Figure 2a). Whereas for cycle 2 (days 113–190) operation, OLR was increased to about 4.7 g.VS/L.d, and the corresponding volumetric combined biogas production was observed to be 7.7 ± 1.8 L/d. The cumulative biogas volume was found to be stable in both cases. As far as the methane concentration in the biogas was concerned, during the cycle 1 operation, it took about 82 days, especially for the HSAD to reach 50% CH4, whereas, in cycle 2, it took only 42 days to attain the same value. Interestingly, methane content in the liquid inoculum reservoirs remained always higher for both the cycles (Figure 2b), which demonstrates that the process offered excellent quality of biogas, which remained fairly steady (70–75%) at the end of each cycle. High methane content also suggested that the methanogenic population in the liquid inoculum reservoirs was enhanced for this substrate (CM + DM mix). It is to be noted that a combined (liquid + HSAB) methane concentration at the end of each cycle had reached to about 70%.

Specific methane yield (SMY) is a parameter that quantifies the amount of methane generation per gram of the organic matter, such as VS or COD. The average SMY was reached to about 0.33 LCH_4_/gVS at the end of cycle 1 operation (i.e., on day 112), whereas a similar result was obtained within 78 days in cycle 2 (Figure 2c). In addition to this, the degradation of organic matter in terms of CODt and VS was monitored. CODt and VS reductions of about 60% and 59%, respectively, were observed at the end of cycle 1. Whereas at the end of cycle 2, CODt and VS removal efficiencies were increased to about 76% and 62%, respectively, even at a shorter cycle length.

From Figure 2d, at a pH range of 7.2–8.4, the alkalinity reached up to 18 g/L in cycle 1 operation and 14 g/L in cycle 2, respectively. A slight change in pH generally could affect the methanogenic activity in AD. However, abrupt changes in pH are balanced by sufficient alkalinity (buffering capacity). Generally, alkalinity generated in the AD system itself controls the system, which is assisted by high protein or nitrogen content in the manure. The levels of VFA remained low (total content below 900 mg/L) at the end of both, indicating high reactor stability, which was confirmed by the presence of more neutralized pH and higher alkalinity values within the digester. There was no sign of inhibition or nutrient deficiency at these operating conditions. The detailed results pertaining to the VFA concentrations are discussed in subsequent sections.

#### 3.2.2. Performance Monitoring of Digesters: Correlation between VFAs, pH, and Methane Concentration

**Figure 3:**
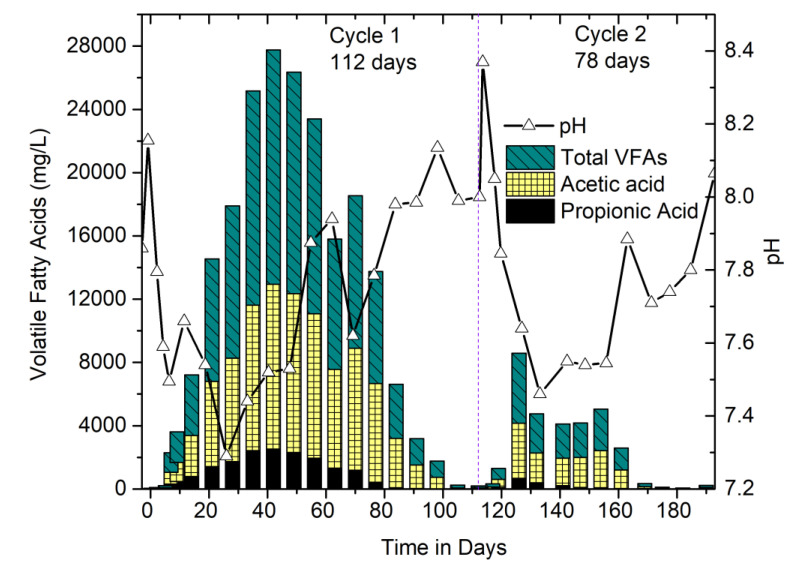
Correlation between volatile fatty acids (VFAs), pH, and methane concentration.

Figure 3 shows the correlation between pH and Total VFA (TVFA). VFAs are the intermediate products in the AD process, and their accumulation is advised to be avoided. The concentration of VFAs is one of the important parameters for the AD process as the increase in VFA indicates the initiation of the acidogenic phase; however, the rapid increase is a sign of inhibition of microorganisms responsible for methanogenesis. Fluctuations in VFA concentration change the pH with the change in hydrogen (H^+^) ions released during the breakdown of organic matter. Maintaining optimal pH is a must for the survival of varieties of microorganisms playing a role in continuing the process without inhibition. The optimal pH range regarded is 6.8–7.2 for both acidogenic and methanogenic bacteria [19]. The pH range in this study was 7.2–8.4, with occasional fall and rise. The initial decrease in pH means the start of acidification, and a sudden increase indicates the termination of acidification at that point. The growth rate of methanogens is slower than the acidogens; therefore, methanogens require longer retention time than the acidogens, in order to consume the VFAs and produce methane-rich biogas. The extraction of VFAs is also possible by providing longer retention time and can be achieved with a batch mode of operation [20]. Low pH leads to the accumulation of acetic acid and hydrogen, which inhibits the degradation of propionic acid and ultimately accumulating VFAs. In the cycle 1 of this study, TVFA production went highest to 15 g/L (42-d), in which 10 g/L was acetic acid, and propionic acid was below 3 g/L. However, the case differed in the next cycle, which only generated a maximum of 4.5 g/L TVFAs (Figure 3). The reason behind comparatively low VFAs could be due to the amount of fatty acids, which declined rapidly due to an appreciable amount of methanogens generated from the previous cycle.

As shown in Figure 2b and Figure 3, between days 51 and 63 of cycle 1, the methane quality was observed to be decreasing (29% on 51-d to 27% on 63-d) in the HSAD digester. Although the decrease was not significant, this could be due to the possibility of scarcity in methanogenic population; therefore, 10 L additional liquid inoculum was recirculated-percolated from liquid inoculum reservoir (digester A) to HSAD (digester B), which then increased the methane concentration due to the increase in their biomass activity. This also facilitated in leaching out the accumulated VFAs from HSAD to the liquid inoculum reservoir for further degradation. Henceforth, after day 65, a significant improvement in methane quality (approximately 45%) in HSAD and a rapid reduction in VFAs (8500 mg/L (66-d) to <200 mg/L (112-d)) in liquid inoculum reservoir were noticed (Figure 3). As far as the cycle 2 operation was concerned, comparatively less feed material was fed along with 50% (*w*/*w*) of the digested material (considered as dry inoculum) from the 1st cycle, which helped to shorten the cycle length with an enriched methane concentration over 50% with minimal VFA accumulations. However, as far as the OLR was concerned, due to the residual COD or VS accumulations from the digested material from cycle 1 operation, there was a slight increase compared to that of cycle 2. In this scenario, the better performance is mostly linked to the adapted microbial populations within the (liquid–solid) system interactions.

#### 3.2.3. Performance Monitoring of Digesters: Ratio Limits

**Figure 4:**
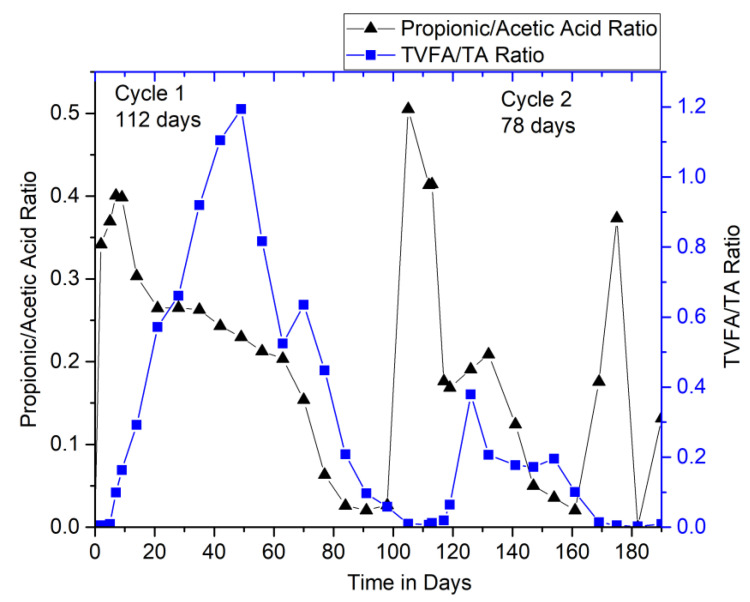
Evolution of TVFA/ TA and propionic acid/acetic acid ratios.

Monitoring the AD process requires proper selection of operational parameters depending upon its metabolic state. The parameters like pH, total alkalinity (TA), temperature, TVFA, and C/N ratio are important as they have a direct influence on the performance of the AD system. The proper understanding of these fundamental parameters and its implementation can exploit the AD to the fullest and avoid the inhibitions that can occur in certain conditions. One of the major factors, which expresses the stability of AD, is the ratio of TVFA/TA, which is reported to be less than 0.5 for high stability [21] and regarded optimal between 0.4 and 0.6 by [22], beyond which is indicated as overfed. Therefore, the profile of these parameters in the digesters during the operation is shown in Figure 4.

Ratio limits like TVFA/TA and propionic acid/acetic acid ratio are the key critical indicators of digester’s crash. Studies suggest TVFA/TA to be less than 1 (preferably within a range of 0.1 and 0.6) [18,19] and propionic acid/acetic acid ratio to be less than 1.4 [23] for the high stability of the digester. In this study, the TVFA/TA ratio remained below 1 in the entire operation period, except for days between 40 and 50 of cycle 1 when it reached up to 1.2. This was an indication of inhibitions due to the lack of active microbes in HSAD. These results were in accordance with the methane concentration profiles (HSAD) (Figure 2b), and henceforth, about 10 L of liquid inoculum was recirculated-percolated to HSAD to overcome this situation. However, the propionic acid/acetic acid ratio reached only up to 0.5 throughout the operation (Figure 4). These indicators, demonstrating that the digesters were operating favorably without the risk of acid-buildup and better stability, attained with time.

#### 3.2.4. Performance Monitoring of Digesters: Relationship between pH, Ammonia (TAN, FAN), and Temperature

**Figure 5:**
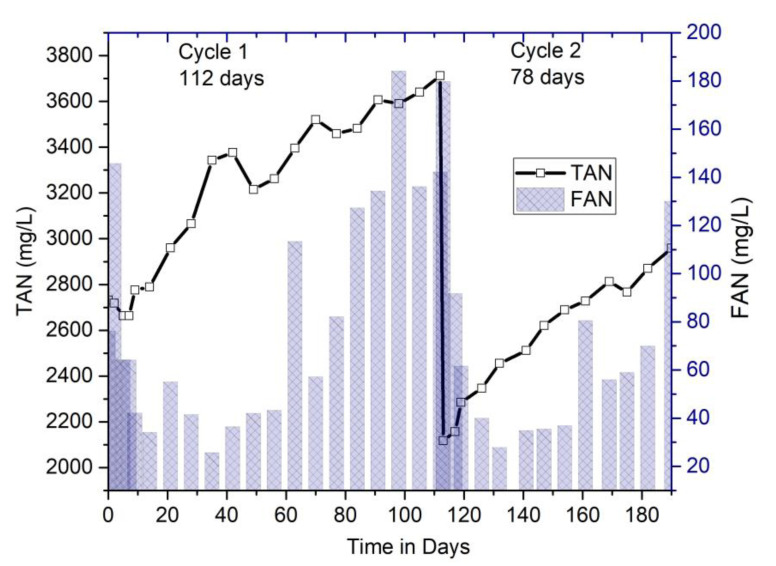
Evolution of total ammonia nitrogen (TAN) and free ammonia nitrogen (FAN) profiles.

The parameters in the AD process are inter-related; thus, an optimal pH and low temperature coupled with high alkalinity balanced the yield of free ammonia in this system. In Figure 5, FAN concentration was shown to be under 200 mg/L in cycle 1 and lower than 180 mg/L in cycle 2. TAN was 3.7 g/L at maximum in the 1st cycle; however, in the 2nd cycle, it was only 3.1 g/L. Generally, exceeding TAN results in the reduction of methane concentration and biogas production. This study, hence, justified the increase in methane-rich biogas with the reduction of TAN.

Around 35–40% of the increase in total ammonia nitrogen (TAN) was observed from the commencement of the operation, and a considerable increment of free ammonia nitrogen (FAN) was also observed.

Ammonia has a significant role in supplying nutrients for microbial growth, maintaining buffering capacity (alkalinity) and stability of the digester. Ammonia is dependent on pH, temperature, alkalinity, and substrates. Ammonia exists in two forms: (i) free ammonia (NH_3_) and (ii) ammonium (NH_4_). Free ammonia is a gas and toxic, and ammonium is in ionized form, which is non-toxic salt. NH_3_ or free ammonia nitrogen (FAN) and NH_4_ together make total ammonia nitrogen (TAN). FAN takes part in the inhibitory actions in the AD process since the high concentration of free ammonia in the system ruptures the cell wall of the microbes, leading to cell lysis. Ammonia is mainly dependent upon temperature and pH mentioned by [24] in Equation (1).

(1) $$\mathrm{FAN}=\mathrm{TAN}\;{\left(1+\frac{10^{-pH}}{10^{-\left(0.09018+\frac{2729.92}{T\left(K\right)}\right)}}\right)}^{-1}$$

where the temperature is in Kelvin (*K*); total ammonia nitrogen (TAN) and free ammonia nitrogen (FAN) are in mg/L.

Microorganisms, which are responsible for the entire AD process, are generally sensitive and survive at certain conditions. Similarly, the temperature is one of the important factors as the growth of the microbes is higher at a higher temperature. However, AD at a temperature > 50 °C is unstable and generates high FAN, especially while treating ammonia-rich wastes, which is an inhibitor for the process itself. FAN is directly proportional to the temperature; therefore, the generation of FAN is lesser at low-temperature conditions, contributing to the fewer chances of AD inhibition. For proper microbial growth at a lower temperature, substrate acclimation at low-temperature conditions is proven to be advantageous [10]. Therefore, in this study, liquid inoculum adapted to 20 ± 1 °C was utilized for the liquid digester in a two-stage operation, which led to the lower generation of FAN of up to 185 mg/L.

**Figure 6:**
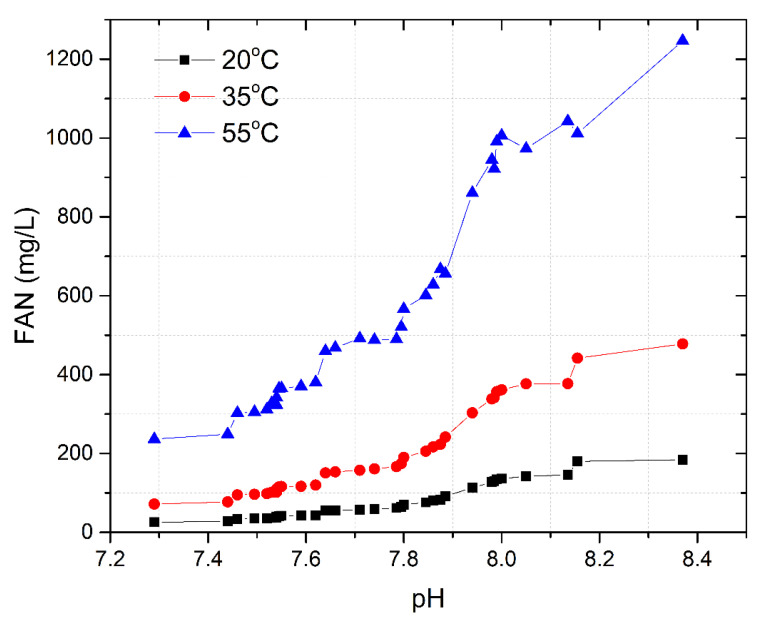
FAN at different temperatures under an operating pH range.

This study also reported the direct proportionality of FAN with different temperatures (for instance, 20 °C, 35 °C, and 55 °C), as shown in Figure 6, based on the formula provided in Equation (1) by extrapolating the concentration of FAN. This was done to derive a theoretical conclusion based on these calculations. Under an operating pH range of 7.2–8.4, FAN at 20 °C was a maximum of 185 mg/L. Extrapolating the results for FAN at different temperatures based on Equation (1) and operating pH range showed that at 35 °C (mesophilic), FAN could have reached up to 500 mg/L, and the same could have ascended to 1300 mg/L at 55 °C (thermophilic). Therefore, a theoretical extrapolation shows the concentration of FAN to be low and balanced at lower temperatures and inhibitory at higher temperatures.

### 3.3. Comparative Study of Two-Stage (Liquid–Solid) AD of CM and Co-Digestion of CM + DM

**Figure 7:**
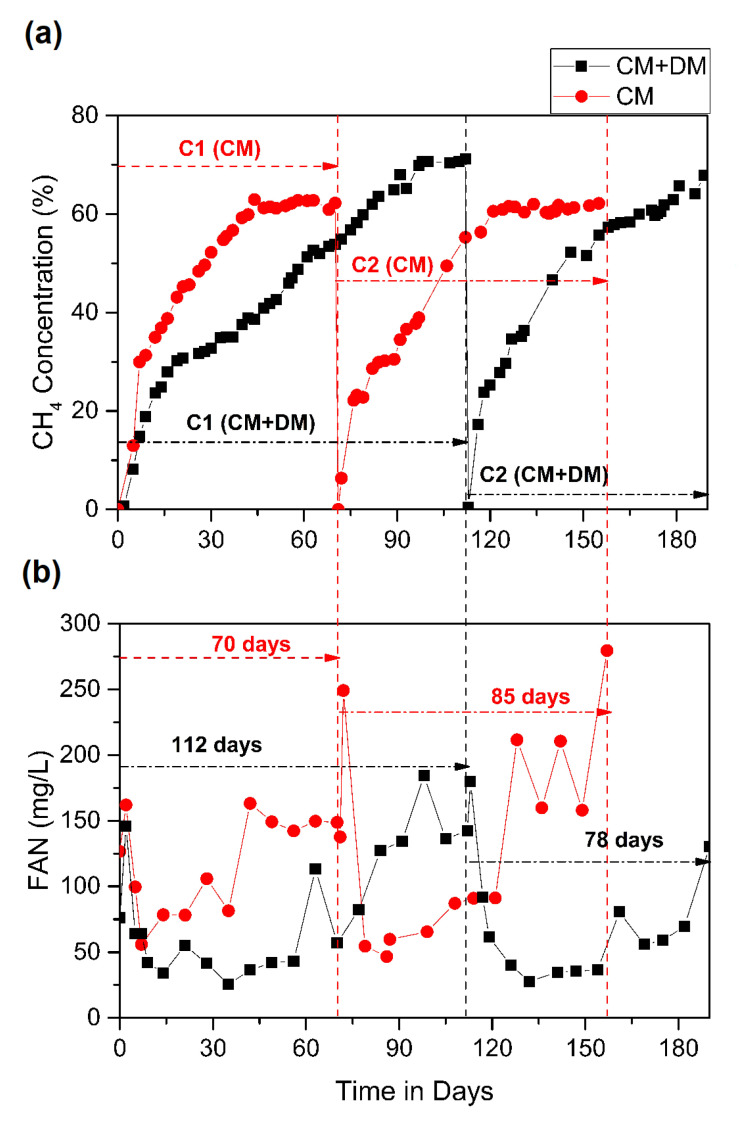
Comparative study of chicken manure (CM) mono-digestion and CM + dairy cow manure (DM) co-digestion: (**a**) Methane concentration profile; (**b**) FAN profile.

The operating conditions of the two-stage (liquid–solid) AD of CM mono-digestion and CM + DM co-digestion are given in Table 2. The digesters were operated in a similar fashion in order to develop a start-up and operating strategies for these substrates. An attempt was made to compare digesters, treating these two substrates in terms of methane yield and its concentrations, and also the release of FAN during the AD processes in order to determine the inhibitory potential of ammonia in the respective feedstocks (Figure 7). The liquid inoculum used to start the digesters for both substrates were adapted to chicken manure leachate. Henceforth, the methane concentrations in cycle 1 of CM mono-digestion showed a quick start-up compared to that of CM + DM co-digestion.

In cycle 1 of both cases, the concentration of methane was approximately 58%. On the contrary to this, in the cycle 2 of CM + DM, on day 78, the CH_4_ concentration was approximately 70%; however, the same for CM was around 60%, making a difference of up to 10% (Figure 7a). These results showed that co-digestion using DM had a positive effect in producing a comparatively better methane-rich biogas. However, methane yield or SMY obtained for the CM mono-digestion was 0.52 ± 0.13 L CH_4_g^−1^VS_fed_, and for CM + DM co-digestion, it was 0.35 ± 0.11 L CH_4_g^−1^VS_fed_ (detailed data not shown). Similarly, the volumetric biogas production was more in CM (13.6 ± 4 L/d) than CM + DM (7.7 ± 1.8 L/d); however, the quality of methane was observed to be better in the co-digestion process. Since the COD_t_:TKN ratio was always higher than 25 for both CM and CM + DM mixture used in this study, better results for CM in terms of methane yield were observed as the CM has a better energy potential than DM.

Furthermore, the release of FAN concentration for both the mono- and co-digestions was monitored to have a better perspective or forecasting of the ammonia inhibition (Figure 7b). It is evident from Figure 7b that FAN concentrations were comparatively lower in the co-digestion process than mono-digestion due to the dilution of higher ammonia content in CM by DM. Contribution to the generation of ammonia not only lies in the initial concentration of the feedstock but also during the biochemical process in AD. CM is high in nitrogen; hence, the initial concentration had a vital role in a higher concentration of FAN than that in CM + DM. On the 70th day of both cases, FAN was 150 mg/L in CM and 50 mg/L in CM + DM. This can be related to the high nitrogen content in CM mono-digestion than the co-digestion, which helped in the dilution of high ammonia. Although there was an increase in the FAN concentration for the mono-digestion of CM, no apparent inhibitions were reported for both the processes during the start-up phase. The probable reason was that the FAN levels were still in the tolerable range (always below 280 mg/L), and the VFA/alkalinity ratio always remained below 0.5 in both the cases, indicating that the digesters were operating favorably without the risk of acid-buildup. Thus, the presence of ammonia nitrogen did not inhibit the performance of the liquid inoculum reservoir, as well as HSAD, even at high OLRs. Even if the pH was not controlled in the bioreactors, there was no formation of foam or scum observed throughout the study. The mode of operation (process, temperature, percolation-recirculation rate, and frequency) and the appropriate choice of acclimatized inoculum at the start-up phase of the experiment allowed a high stabilization of CM + DM co-digestion, even at higher OLR (4.7 gVS/L.d) studied. Further study is underway to optimize the operating parameters, especially for the co-digestion process.

## 4. Conclusions

The proposed start-up study focused on two-stage (liquid inoculum reservoir coupled with HSAD) anaerobic digestion process using a closed-loop recirculation, and percolation mode operation was found efficient for the treatment of CM + DM at 20 ± 1 °C despite having a waste mix with high TKN (13.5–13.6 gN/L) and solid (TS: 48–51%) concentrations. Results showed that our system could generate a specific methane yield of 0.35 ± 0.11 L CH_4_g/VS_fed_ at an OLR of 3.7–4.7 gVS/L.d. We also observed CH_4_ concentrations above 60% with CODs and VS reduction by up to 85% and 60%, respectively. A comparative study was done using the same start-up protocol to perform the mono-digestion of CM (TKN: 21–23 g/L; TS: 65–73%). Although a better SMY (0.52 ± 0.13 L CH_4_g^−1^VS_fed_) was obtained for mono-digestion of CM, co-digesting CM + DM showed a better methane quality and also generated comparatively lower FAN. However, no evident inhibitions due to ammonia or VFA accumulations were reported for both the processes during the start-up phase. Compared to the higher-temperature digestion process, more energy is expected to be available for farm uses, especially while treating high solids and ammonia-rich wastes.

## Figures and Tables

**Table 1:** Summary of the materials used.

	Cycle 1	Cycle 2
Total weight of feedstock treated	7 kg (CM + DM)	4.7 kg (CM + DM)+ 4.7 kg (Dry inoculum from cycle 1)) = 9.4 kg
Quantity increment (%) per cycle	-	34% w/w
Mix ratio (CM:DM)	1:1	1:1
Volume of liquid inoculum	25 L	25 L
Solid substrate: liquid inoculum digester volumetric ratio	1:3.6	1:2.6
OLR (gVS/L.d) *	3.7	4.7

* OLR calculations were done based on the raw feedstock VS, and the formula used was OLR = VSi * (Q/V), where OLR: organic loading rate (g VS/L.d); VSi: VS of feedstock (CM + DM) in g/L; Q: quantity/flow rate of raw feedstock in kg or L/d; V: volume of the HSAD in L.

**Table 2:** Operating conditions of mono-digestion (CM) and co-digestion (CM + DM).

	CM (C1)	CM + DM (C1)	CM (C2)	CM + DM (C2)
**Cycle length (retention time or treatment period)**	70	112	85	78
**Quantity of raw manure treated (kg)**	5.4	7	6.5	4.7
**Total volume of HSAD (L)**	60	40	60	40
**Total amount of solid material treated in HSAD (kg)**	10	7	10.8	9.4
**Total volume of liquid digester (L)**	60	40	60	40
**Active volume of liquid digester (L)**	25			
**Quantity and frequency of liquid inoculum percolated-recirculated**	5L-thrice a week			
**Mode of operation**	Batch			
**Temperature (°C)**	20 ± 1			
**OLR (gVS/L.d)**	4.3	3.7	4.6	4.7

OLR = organic loading rate; CM = chicken manure; DM = dairy cow manure; C1 = cycle 1; C2 = cycle 2.

**Table 3:** Characteristics of feedstock and inoculum.

Parameter	Cycle 1				Cycle 2			
	CM	DM	Inoculum	CM + DM	CM	DM	Inoculum	CM + DM
**pH**	8.68	7.58	7.86	8.2	8.88	8.13	8.37	8.1
**COD_t_ (mg/L)**	568,017	208,433	7121	405,534	565,885	188,341	5968	402,921
**COD_s_ (mg/L)**	114,768	44,852	4415	94,044	111,545	34,017	3915	96,944
**Alkalinity (as** **mg/L CaCO_3_)**	33,282	13,932	13,313	12,649	30,486	11,126	9575	-
**TS (%)**	65	23.9	1.28	48	73	21.58	1.02	51
**VS (%)**	56	21.3	0.54	42	65	19.23	0.40	45
**TKN (mg/L)**	21,962	6749	3151	13,613	23,072	5194	2359	13,472
**NH_3_ (mg/L)**	6070	1389	2732	3470	7229	1795	2117	-
**TVFA (mg/L)**	11,588	6973	24	10,582	10,914	6499	116	-
**CODt/TKN**	25.8	31	2	30	25	36	3	30

## References

[1] High Level Expert Forum—How to Feed the World in 2050. http://www.fao.org/fileadmin/templates/wsfs/docs/Issues_papers/HLEF2050_Global_Agriculture.pdf.

[2] AVEC (Association of Poultry Processors and Poultry Trade in the EU Countries) Annual Report 2016. http://www.avec-poultry.eu/wp-content/uploads/2018/04/AVEC-2016-BAT.pdf.

[3] Fatma A., Namba N., Kesseva M.R., Nishio N., Nakashimada Y. (2014). Enhancement of methane production from co-digestion of chicken manure with agricultural wastes. Bioresour. Technol..

[4] MacLeod M., Gerber P., Mottet A. (2012). Greenhouse Gas Emissions from Pig and Chicken Supply Chains.

[5] Werner F., Wang X., Gabauer W., Ortner M., Li Z. (2018). Tackling ammonia inhibition for efficient biogas production from chicken manure: Status and technical trends in Europe and China. Renew. Sustain. Energy Rev..

[6] Intergovernmental Panel on Climate Change (IPCC) (2001). Climate Change 2001: The Scientific Basis.

[7] Sun X., Lu P., Jiang T., Schuchardt F., Li G. (2014). Influence of bulking agents on CH_4_, N_2_O, and NH_3_ emissions during rapid composting of pig manure from the Chinese Ganqinfen system. J. Zhejiang Univ. Sci. B.

[8] Lynch D., Henihan A.M., Bowen B., Lynch D., McDonnell K., Kwapinski W. (2013). Utilisation of poultry litter as an energy feedstock. Biomass Bioenergy.

[9] Kelleher B.P., Leahy J.J., Henihan A.M., O’Dwyer T.F., Sutton D., Leahy M.J. (2002). Advances in poultry litter disposal technology—A review. Bioresour. Technol..

[10] Rajinikanth R., Massé D.I., Singh G. (2013). A critical review on inhibition of anaerobic digestion process by excess ammonia. Bioresour. Technol..

[11] Suleyman K.Y.S., Kocak E. (2009). Anaerobic digestion technology in poultry and livestock waste treatment—A literature review. Waste Manag. Res..

[12] Matheri A.N., Ndiweni S.N., Belaid M., Muzenda E., Hubert R. (2017). Optimising biogas production from anaerobic co-digestion of chicken manure and organic fraction of municipal solid waste. Renew. Sustain. Energy Rev..

[13] Singh K., Lee K., Worley J., Risse L.M., Das K.C. (2010). Anaerobic digestion of poultry litter: A review. Appl. Eng. Agric..

[14] Siddique M.N.I., Munaim M.S.A., Wahid Z.A. (2014). Mesophilic and thermophilic biomethane production by co-digesting pretreated petrochemical wastewater with beef and dairy cattle manure. J. Ind. Eng. Chem..

[15] Li R., Chen S., Li X., Lar J.S., He Y., Zhu B. (2009). Anaerobic co-digestion of kitchen waste with cattle manure for biogas production. Energy Fuels.

[16] Eaton A.D., Clesceri L.S., Rice E.W., Greenberg A.E., Franson M.A.H.A. (2005). APHA: Standard Methods for the Examination of Water and Wastewater.

[17] Massé D.I., Masse L., Croteau F. (2003). The effect of temperature fluctuations on psychrophilic anaerobic sequencing batch reactors treating swine manure. Bioresour. Technol..

[18] Esposito G., Frunzo L., Giordano A., Liotta F., Panico A., Pirozzi F. (2012). Anaerobic co-digestion of organic wastes. Rev. Environ. Sci. Bio Technol..

[19] Khanal S.K. (2011). Anaerobic Biotechnology for Bioenergy Production: Principles and Applications.

[20] Li H.L., Guo X.L., Cao F.F., Wang Y. (2014). Process evolution of dry anaerobic co-digestion of cattle manure with kitchen waste. Chem. Biochem. Eng. Q..

[21] Rajagopal R., Ramirez I., Steyer J.P., Mehrotra I., Kumar P., Escudie R., Torrijos M. (2008). Experimental and modeling investigations of a hybrid upflow anaerobic sludge-filter bed (UASFB) reactor. Water Sci. Technol..

[22] Brown D., Li Y. (2013). Solid state anaerobic co-digestion of yard waste and food waste for biogas production. Bioresour. Technol..

[23] Ehimen E.A., Sun Z.F., Carrington C.G., Birch E.J., Eaton-Rye J.J. (2011). Anaerobic digestion of microalgae residues resulting from the biodiesel production process. Appl. Energy.

[24] Hansen K.H., Angelidaki I., Ahring B.K. (1998). Anaerobic digestion of swine manure: Inhibition by ammonia. Water Res..

